# Preparation and Hg^0^ Removal Performance of MIL-101(Cr)-Derived Carbon Matrix Composites

**DOI:** 10.3390/polym17030413

**Published:** 2025-02-04

**Authors:** Haotian Nie, Zikuo Li, Xikai Zhang, Jinchao Wen, Youxiang Feng, Yue Yu, Li Jia

**Affiliations:** 1 College of Electrical and Power Engineering, Taiyuan University of Technology, Taiyuan 030024, China; niehaotian0327@163.com (H.N.); 19834008532@163.com (Z.L.); zxklanyi001@163.com (X.Z.); 18835003405@163.com (J.W.); 15734969860@163.com (Y.F.); 2 College of Economics and Management, Taiyuan University of Technology, Taiyuan 030024, China; yuyue@tyut.edu.cn

**Keywords:** MIL-101(Cr), biochar, composites, mercury removal

## Abstract

The temperature at which pollutants are treated varies across different industrial processes. To address the high cost of raw materials for MOFs and the low efficiency of Hg^0^ removal in low-temperature environments, a series of MIL-101(Cr)-derived carbon matrix composite materials were prepared by combining MIL-101(Cr) with biomass and multiple metals. These materials were synthesized through a sol-gel method followed by carbonization. This study investigates the effects of composite ratios and adsorption temperatures on Hg^0^ removal, utilizing XRD, BET, and other characterization techniques to elucidate the mercury-removal mechanism of the PDC-MIL composite materials. The results indicate that MIL101(Cr) significantly influences the formation of the gel skeleton. When the composite ratio of MIL-101(Cr) to biomass is 1:1, the material exhibits an optimal pore structure, leading to high Hg^0^ removal efficiency over a wide temperature range. The removal of Hg^0^ by these composite materials involves both physical adsorption and chemisorption. Low temperatures favor physical adsorption, while high temperatures promote chemisorption. The sol-gel composite method facilitates cross-linking polymerization between MOFs and SiO_2_, enabling better pore structure connectivity with biomass and MOFs, thereby optimizing the poor pore structure observed after pyrolysis. Consequently, the improved pore structure enhances physical adsorption at low temperatures, mitigates desorption at high temperatures, and increases the contact probability of Hg^0^ with active sites within the pores, significantly improving the mercury-removal ability of the material across a broad temperature range.

## 1. Introduction

Mercury (Hg) pollution poses a serious threat to the environment and human health, and has a biomagnification effect in ecosystems, leading to increasing global concern about mercury pollution. As the main source of anthropogenic mercury emission, the mercury in the smoke gas of coal-fired power plants mainly exists in three forms: oxidized mercury (Hg^2+^), particulate mercury (Hg^P^) and elemental mercury (Hg^0^) [1]. The oxidized mercury (Hg^2+^) and particulate mercury (Hg^P)^ can be removed by the existing wet flue gas desulfurization (WFGD) and air pollutant control device (APCDs) in thermal power plants, while the elemental mercury (Hg^0^) is difficult to remove due to its insolubility in water [2]. Therefore, the control and removal of Hg^0^ has become the focus of difficulty in mercury removal from flue gas.

At present, coal-fired power plants do not have specialized mercury emission control devices, so the adsorbent injection method used in conjunction with existing flue gas purification equipment has become a high-concern mercury-removal technology. Direct adsorption of elemental mercury (Hg^0^) by adsorbents has been widely adopted at the industrial scale. The process involves injecting adsorbents into coal flue gas, capturing Hg^0^ via physical or chemical interactions and subsequently separating it using APCDs. Currently, activated carbon injection (ACI) is recognized as the most mature and feasible technology for mercury emission control in coal-fired power plants [3]. Activated carbon (AC) exhibits superior mercury-adsorption performance because of its high specific surface area and abundant surface oxidizing groups. Studies have shown that surface modification of AC with halogens and metal oxides can effectively improve its mercury-adsorption efficiency [4]. Nevertheless, the cost associated with mercury removal using AC is relatively high, and the addition of AC increases the carbon content in fly ash, thus affecting its comprehensive utilization. Compared with ACI technology, the fixed-bed pollutant removal technology offers the advantage of the simultaneous removal of multiple pollutants, conserves energy and resources, reduces secondary pollution, and improves economic benefits. However, the selection, regeneration, stability, and cost of adsorbents used in fixed beds pose challenges that limit their application in coal-fired power plants. Therefore, the development and utilization of efficient and cost-effective adsorbents have become priorities. In order to overcome the shortcomings of activated carbon adsorbents and achieve more efficient removal of mercury, a variety of new mercury adsorbents, such as metal oxides, modified fly ash, and metal–organic frameworks (MOFs), have been extensively studied [5].

Metal–organic frameworks (MOFs) are a new type of highly ordered porous materials formed by the self-assembly of inorganic metal ions or clusters and organic ligands through coordination bonds [6]. Compared with traditional adsorbents, MOFs have high specific surface area, porosity, and adjustable structure, making them widely used in adsorption, storage, separation, and other fields. The highly dispersed unsaturated metal sites in the MOFs’ skeleton also make them show high activity in some catalytic reactions. In addition, the chemical tunability of MOFs allows for the functional adjustment of physical and chemical properties through targeted structural design. Among them, MIL-101(Cr) has a high specific surface area (about 2500 m^2^/g) and good thermal stability, and because Cr is rich in valence states (Cr^2+^, Cr^3+^, Cr^5+^, Cr^6+^), MIL-101(Cr) has better chemical activity than other MOFs [7]. It is an ideal material for mercury adsorption. Zhao et al. [8] studied the adsorption characteristics of MIL-101 (Cr) on Hg^0^ and found that MIL-101 (Cr) exhibited better mercury-removal performance compared to Cu-BTC, UiO-66, and activated carbon. Moreover, its mercury-removal efficiency gradually increased with the increase in adsorption temperature, reaching 88% at 250 °C.

However, the removal efficiency of mercury from MOFs at low temperatures is very low. Because the pore structure of MOFs is mainly composed of micropores, the smaller pores hinder the diffusion process of Hg^0^ inside the material. On the one hand, the physical adsorption of Hg^0^ to the material is limited, and on the other hand, the contact process in the Mars–Maessen mechanism is limited. Existing studies have shown that MOFs-derived carbon materials produced from MOFs’ carbonization can improve the mercury-removal performance in the lower temperature range. Zhu et al. [9] obtained derived carbon materials by directly pyrolyzing MIL-101(Cr) and found that the derived carbon materials retained the original skeleton structure of MOF and the active sites were also exposed. The mercury-removal capacity of 100% was achieved in a wide temperature range (30–200 °C), and after continuous adsorption at 30 °C for 50 h, the removal efficiency of mercury remained above 90%. However, the use of single MOFs to produce the derived carbon materials has a high cost, lacks rich functional groups, and is easy to lead to metal ions or clusters oxidation–reduction to metal nanoparticles during carbonization. With the deepening of carbonization, the metal nanoparticles will migrate and merge, and metal agglomeration will occur, thus affecting the mercury-removal performance of the materials. In order to solve the above problems, the combination of MOFs and various functional materials has become a research hotspot. At present, the composite of MOFs and carbon nanotubes, graphene oxide, biomass, and other substances has been achieved, and a series of composite materials with high-efficiency adsorption, stability, and recycling ability have been successfully prepared [10].

Biomass materials have emerged as a research focus due to their renewable, degradable, and abundant nature, as well as their wide availability [11]. Biochar, produced by the pyrolysis of biomass, is a carbon-based material similar to activated carbon, characterized by its developed pore structure and rich surface functional groups. The use of biochar to remove elemental mercury from flue gas has been widely studied. Zeng et al. [12] modified peanut shell biochar using halogen ions and metal oxides to investigate its mercury-removal performance. The results showed that the modified biochar has an abundant pore structure and uniform distribution of the surface-active components, achieving a cumulative mercury-adsorption capacity of 5587 μg/g within 24 h. Jia et al. [13] found that modifying forestry waste walnut shell with multi-metal doping followed by carbonization significantly improved the mercury-removal performance of biochar, effectively achieving waste decontamination. Based on these characteristics of biomass, integrating MIL-101(Cr) with biomass materials can yield MOFs-derived carbon matrix composites, enhancing mercury-removal efficiency and enabling low-cost preparation of an effective adsorbent.

In summary, in order to improve the pain points of a single MOFs adsorbent with narrow temperature range, poor stability, and high cost, in this paper, based on the physical and chemical characteristics of biomass and the basic characteristics of MOFs containing unsaturated metal centers, we combined MIL-101(Cr) with biomass via the sol-gel method and doped with multiple metals and then carbonized to obtain a series of MOFs-derived carbon matrix composites. In addition, on the basis of determining the best composite ratio, the effect of the incorporation of biomass on the removal of mercury from MOFs-derived carbon adsorbents and its mechanism were investigated, which provided a new way for the preparation of wide temperature range, high performance, and low cost of mercury-removal adsorbents.

## 2. Materials and Methods

### 2.1. Preparation of Samples

#### 2.1.1. MIL-101(Cr)

MIL-101(Cr) was prepared by solvent-thermal method [14]. In total, 4 g (10 mmol) Cr(NO_3_)_3_•9H_2_O and 1.66 g (10 mmol) terephthalic acid were dissolved in 40 mL deionized water, then 0.4 mL of 40% HF was added, and the mixture was evenly stirred by a magnetic stirrer for 1 h at room temperature. The obtained suspension was placed in a hydrothermal synthesis reactor lined with polytetrafluoroethylene and reacted in an oven at 205 °C for 20 h. The obtained green solid was centrifuged, washed with DMF and anhydrous ethanol several times, and dried in an oven at 150 °C for 24 h to obtain MIL-101(Cr).

#### 2.1.2. Preparation of Modified Biochar (MBC)

Based on the previous research results [15], walnut shell biomass was used as the matrix, and after multi-metal modification, its mercury-removal effect is excellent. China’s walnut shell production is rich, ranking first in the world. The walnut shell was ground into powder, and the walnut shell powder with a particle size ranging from 58 μm to 75 μm was selected as the matrix. The mass of the compound of multi-metal elements was obtained according to the following formula:

(1) $$m_{c}=\frac{15}{0.84}w\left(A\right)\frac{M_{c}}{M_{A}}$$

In the formula, $m_{c}$ is the mass of the compound of A needed, $w\left(A\right)$ is the mass fraction of the element A needed to be incorporated, $M_{A}$ is the molar mass of element A, and $M_{c}$ is the molar mass of the compound of element A, both of which are g/mol.

According to the calculation results of the formula, 15 g walnut shell powder, 2.214 g Ce(NO_3_)_3_•6H_2_O, 8.643 g FeCl_3_•6H_2_O, 1.403 g CuSO_4_•5H_2_O were dissolved in a mixed solution of 100 mL anhydrous ethanol and 20 mL deionized water. It was stirred well until the bottom of the cup had no obvious granules. Then 1 mL DMF and 15 mL 1, 2 propylene oxide were added, and it was stirred evenly to form a sol-gel state, heated it in a water bath at 40 °C for 24 h, and then a mixture of 2.7 mL ethyl orthosilicate and 0.7 mL anhydrous ethanol was slowly added to the stirring sol. After stirring evenly, it was heated in a water bath at 60 °C for 24 h, dried at 90 °C, and then taken out for grinding. The obtained powder was pyrolyzed at 600 °C and in a N_2_ atmosphere for 10 min to obtain 10%Fe-4%Ce-2%Cu-modified biochar.

#### 2.1.3. Preparation of MIL-101(Cr)-Derived Carbon Materials (PDC-MIL) and Its Composite Materials (PDC-MIL/BM)

**Figure 1:**
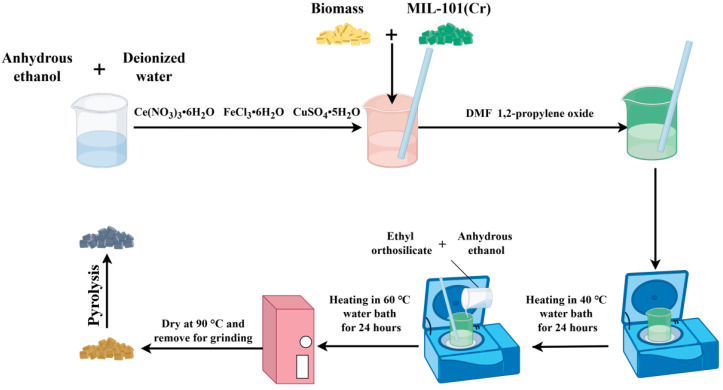
The synthesis roadmap of MIL-101(Cr)-derived carbon matrix composites.

The porous derived carbon material, named PDC-MIL, was prepared after 1 g MIL-101(Cr) was put into a tube furnace at 600 °C, and nitrogen was continuously pumped into the furnace at a flow rate of 1 L/min and pyrolyzed for 10 min. The composite materials were prepared using the sol-gel method, specifically by referring to the preparation process of modified biochar. MIL-101 (Cr) was poured into the mixed solution containing walnut shell powder and metal ions and stirred evenly before the addition of DMF and 1,2-propylene oxide, and then prepared according to the preparation process of modified biochar. According to the mass ratio of MIL-101(Cr) to biomass and to keep the mass fraction of doped metal unchanged, the name of the composite material was recorded as PDC-MIL/BM (1:5, 1:3, 1:1, 3:1, 5:1). The synthesis roadmap of MIL-101(Cr)-derived carbon matrix composites is shown in Figure 1.

### 2.2. Experimental System and Characterization Method

**Figure 2:**
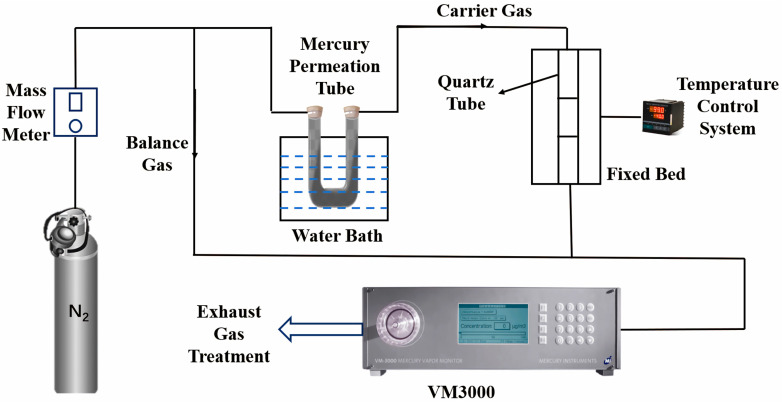
Fixed-bed mercury-adsorption experiment system.

The mercury-adsorption properties of MIL-101(Cr) and PDC-MIL/BM composites were evaluated through a fixed-bed mercury removal experiment, as shown in Figure 2. Elemental mercury was produced by heating the mercury permeation tube in a water bath. The concentration of Hg^0^ at the inlet of the reactor is adjusted by controlling the temperature of the water bath. The elemental mercury produced was carried by carrier gas into the fixed-bed reactor. The mercury concentration at the outlet of the fixed bed was measured using the VM3000 mercury continuous on-line monitor produced by MI Company of Germany, and the Hg^0^ concentration was monitored every 1 s and recorded by the computer. The amount of adsorbent was 0.1 g, which was loaded on the porous quartz plate in the center of the quartz tube. In order to meet the requirements of VM3000(MI, North Rhine, Westphalia, Germany) for intake, the total gas flow during the experiment was set at 1.4 L/min, in which the carrier gas flow is 500 mL/min and the balance gas flow introduced from the bypass is 900 mL/min. The adsorption temperature during the experiment was controlled by the tube furnace at 50 °C, 100 °C, 150 °C, 200 °C, and 250 °C, respectively. The tail gas produced during the experiment was treated with KI-modified coconut shell activated carbon.

The cumulative mercury-adsorption capacity per unit mass of the sample, denoted as *q*, is used as the evaluation index for the material’s mercury-removal performance, and the calculation formula is as follows:

(2) $$q=\frac{F}{m}\sum_{i=0}^{t}\left(C_{\mathrm{in}}-C_{\mathrm{out},i}\right)$$

where q is the accumulated mercury-adsorption capacity, ng/g; F is the flow rate, L/s; m is the sample mass, g; $C_{\mathrm{in}}$ and $C_{\mathrm{out},i}$ are, respectively, the inlet mercury concentration of the reactor and the outlet mercury concentration at time i, ng/L; and t is the adsorption time, s.

In this paper, the crystal-phase structure of the sample was characterized by a BRUKER D8 ADVANCE series X-ray diffractometer (BRUKER, Karlsruhe, Germany), and the scanning speed was 10°/min. The pore structure of the sample was analyzed by MICROMERITICS ASAP2460 specific surface and porosity analyzer (Micromeritics, Norcross, GA, USA). FT-IR characterization with Nicolet iS20 (Thermo Fisher, Waltham, MA, USA) was used to analyze the chemical functional groups on the sample surface.

## 3. Results and Discussion

### 3.1. Crystalline Structure

**Figure 3:**
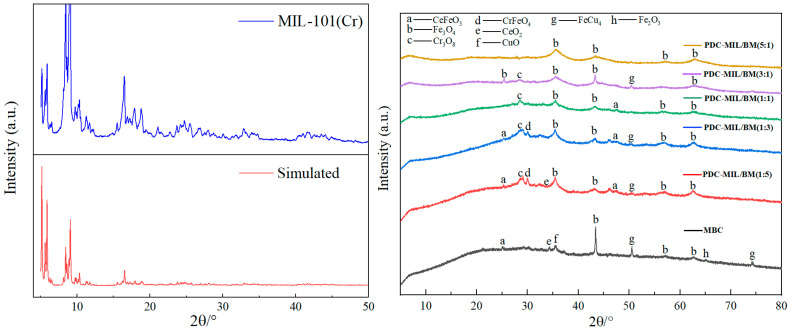
XRD patterns of MIL-101 (Cr) and its composite materials.

The crystal structure of the adsorbent was elucidated using XRD analysis, as illustrated in Figure 3. MBC predominantly exhibits diffraction peaks for oxides of Fe, Ce, and Cu, along with their solid solutions, which increase the number of active sites on biochar, thereby augmenting its adsorption capabilities [16]. The XRD pattern of MIL-101(Cr) aligns with the documented references [17], confirming the successful synthesis of the MIL-101(Cr) material. The XRD of the PDC-MIL/BM material obtained after pyrolysis is shown in Figure 3. It can be seen from the figure that the diffraction characteristic peaks of MIL-101(Cr) basically disappear. At an MIL-101(Cr) to biomass doping ratio of 5:1, the pyrolyzed material predominantly displays the diffraction peaks of Fe_3_O_4_, indicating that during preparation, Fe^3+^ ions infiltrate the pores and surfaces of MOFs and biomass. During the pyrolysis process, the MOF framework collapses, and the ligand terephthalic acid decomposes, forming an oxygen source of metal oxides together with oxygen-containing functional groups in biomass. Due to its stronger oxophilicity, Fe^3+^ more readily binds with these oxygen-containing groups to form metal oxides. When the doping ratio is 5:1, the limited oxygen-containing functional groups on the surface due to the lower biomass content result solely in the diffraction peak of iron oxide. As the ratio of biomass doping increases, the swelling peak of carbon material becomes more pronounced in the low-angle region, reaching its maximum at a ratio of 1:5, and displays the characteristic peaks of various metal oxides. It can be seen that the addition of biomass not only supplies rich carbon sources, to improve the porosity of composite materials, but also provides abundant oxygen-containing functional groups, facilitating the formation of metal oxides. The scant diffraction characteristic peaks of chromium oxides may be attributed to the adherence of metal ions to the surface of MIL-101(Cr) during the doping process. This adherence likely leads to the encapsulation of both MIL-101(Cr) and the formed chromium oxides during metal oxide synthesis. Furthermore, due to the brief duration of the pyrolysis time, most of the MIL-101(Cr) framework remains uncollapsed, resulting in composites characterized by metal oxides of iron, cerium, and copper encasing both MIL-101(Cr) and some chromium oxides.

### 3.2. Pore Structure

**Figure 4:**
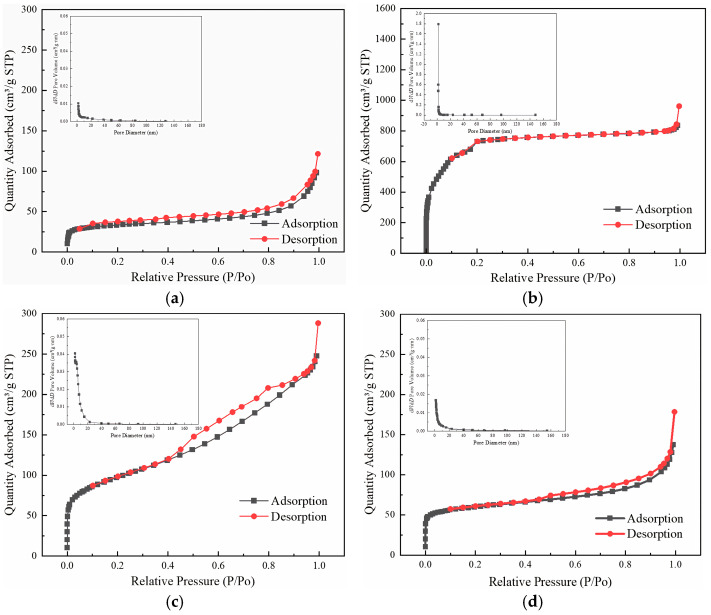

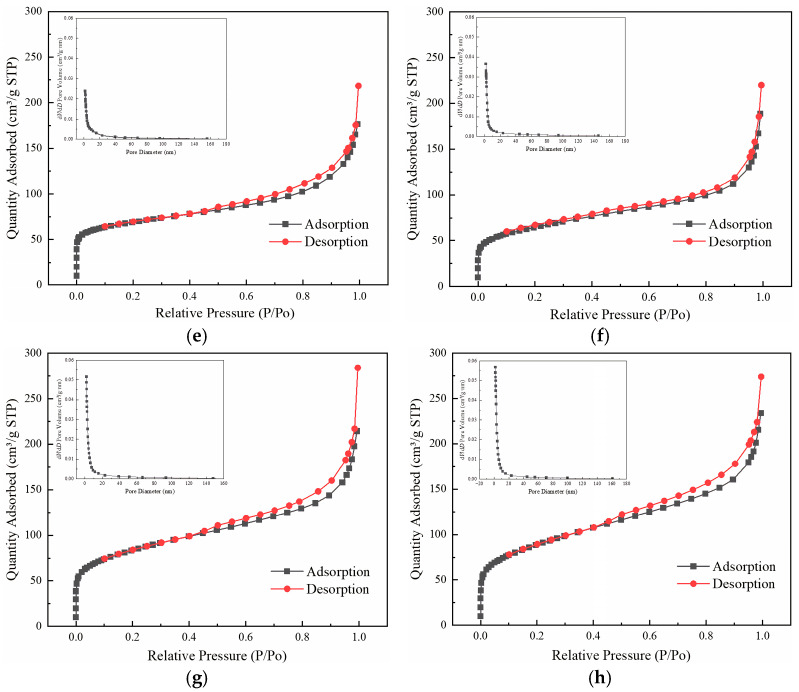
N2 adsorption–desorption isothermal curves and pore size distribution diagrams. (**a**) MBC, (**b**) MIL-101(Cr), (**c**) PDC-MIL, (**d**) PDC-MIL/BM (1:5), (**e**) PDC-MIL/BM (1:3), (**f**) PDC-MIL/BM (1:1), (**g**) PDC-MIL/BM (3:1), (**h**) PDC-MIL/BM (5:1).

The pore structure and specific surface area of MBC, PDC-MIL, their composites PDC-MIL/BM (1:5, 1:3, 1:1, 3:1, 5:1), and unpyrolyzed MIL-101(Cr) were investigated by N_2_ adsorption and desorption experiments. As shown in Figure 4c, the hysteresis loop configuration of the pyrolyzed MIL-101(Cr) material, PDC-MIL, predominantly exhibits irregularity. This irregularity could stem from diverse pore structures in the sample or the interconnected pores channels, leading to complex adsorption behavior. Upon combining MIL-101(Cr) with biomass, the material’s adsorption and desorption curves displayed a distinct H3 hysteresis loop, which was mainly attributed to the sheet-like granular carbon material produced in the pyrolysis process of biomass. With the increase in the proportion of MOFs, the hysteresis loops gradually disappear and completely disappear at a 1:1 ratio. However, with a further increase in the MOFs’ ratio, H3 hysteresis loops gradually appear, mainly because of the structural collapse of MOFs during the pyrolysis process, transforming most micropores into irregular mesopores. When the ratio of the two is 1:1, the adsorption and desorption curves of the composite material align closely, indicating that the material exhibits good pore uniformity. There exists a synergistic effect between the pores of MOFs and biochar. The combination of the sheet-like pores of biochar and the irregular mesopores of MOFs combine to efficiently optimize the pore structure. It can be seen from the pore size distribution diagrams that compared with the MIL-101(Cr), the mesoporous proportion in pyrolyzed PDC-MIL greatly increases, transitioning from a microporous to a mesoporous material. Therefore, the microporous proportion of the composite material decreases while its mesoporous proportion increases, which is more favorable for the transfer of Hg^0^ with larger atomic size within the material. This transformation enhances the adsorption properties.

The pore structure parameters of the materials are summarized in Table 1. The specific surface area of MIL-101 (Cr) exceeds 2400 m^2^/g, which aligns with previously reported literature values and confirms the successful synthesis of MIL-101 (Cr). This material has a microporous proportion of 60.88%, classifying it as a typical microporous material. However, both the pyrolysis process and the incorporation of biomass substantially reduce the BET-specific surface area of the composite materials, leading to a predominance of mesopores and macropores. Specifically, pyrolysis induces the collapse of the internal pore structure of MIL-101 (Cr), transforming a significant portion of micropores into mesopores, increasing from 29.68% pre-pyrolysis to 84.89%. Additionally, biomass forms sheet-like mesoporous carbon particles during pyrolysis, further contributing to the high proportion of mesopores and macropores observed in MBC. As the proportion of MIL-101 (Cr) increases, the specific surface area of the composite materials gradually rises, along with an increase in the micropore proportion. Notably, the mesoporous proportion peaks at a 1:1 ratio but decreases as the MIL-101 (Cr) content continues to rise, while the micropore proportion increases significantly. In the adsorption of Hg^0^, micropores provide adsorption sites, mesopores facilitate the diffusion of Hg^0^ within the material, allowing Hg^0^ to penetrate deeper into the adsorbent, and macropores can reduce the pressure drop inside the adsorbent, which is beneficial for the regeneration and long-term use of the adsorbent [18]. Therefore, an optimal ratio of micropores to mesopores can effectively improve the adsorption efficiency of Hg^0^. It has been observed that PDC-MIL/BM (1:1) can promote both surface adsorption and efficient internal diffusion of Hg^0^ due to its well-balanced micropore and mesopore ratio. Its uniform pore distribution also facilitates contact between Hg^0^ and active sites. Pore volume and average pore size are important parameters for evaluating the richness of a material’s pore structure. A combination of smaller pore sizes and larger pore volumes indicates greater pore abundance. According to Table 1, MIL-101 (Cr) demonstrates the highest pore abundance; however, its high proportion of micropores limits its effectiveness in achieving efficient adsorption at low temperatures. In contrast, PDC-MIL in carbon materials not only possesses the highest pore abundance but also features a significant proportion of mesopores, making it highly promising for low-temperature adsorption.

### 3.3. Surface Chemical Characteristics

**Figure 5:**
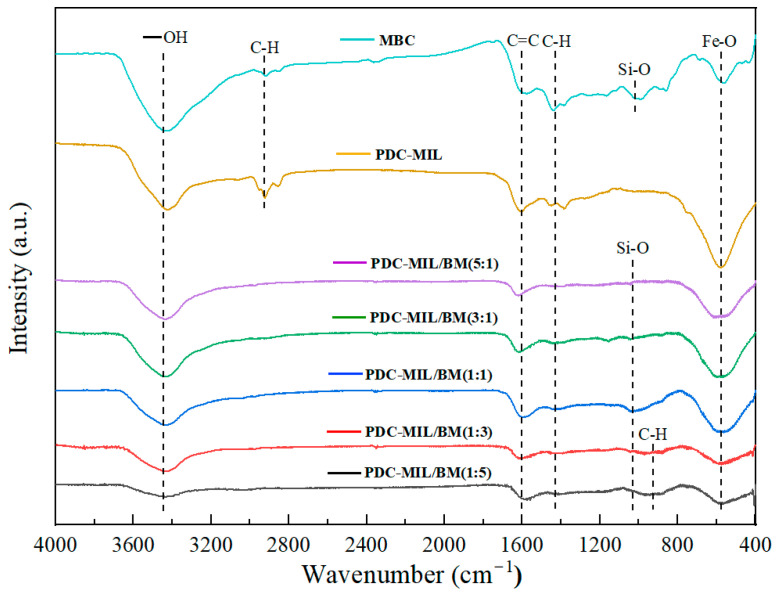
Infrared spectrum of composite materials.

The FTIR spectra of various materials is shown in Figure 5, where 3436 cm^−1^ corresponds to the stretching vibration of -OH, 3000 cm^−1^ corresponds to the stretching vibration of the aliphatic compounds -CH_2_ and -CH_3_, 1612 cm^−1^ corresponds to the stretching vibration of the C=C double bond, 1420 cm^−1^ corresponds to the in-plane bending vibration of C-H, and 571 cm^−1^ corresponds to a strong absorption peak related to the stretching vibration of Metal-O. Specifically, PDC-MIL is linked to the Cr-O bond, MBC to the Fe-O bond, and the composite materials display an overlay of the Cr-O and Fe-O bonds. With the increase in the proportion of MOFs and the decrease in the proportion of biomass, the C-H out-of-plane bending vibration absorption peak at 930 cm^−1^ weakens and disappears when the ratio reaches 1:1. Due to the addition of ethyl orthosilicate, the absorption peak of the Si-O bond appears at 1037 cm^−1^, with increased intensity at an MIL-101(Cr) to biomass ratio of 1:1, and at other ratios, the absorption peak of the Si-O bond was smaller, significantly weaker than 1:1. This indicates that SiO_2_ is well dispersed between the MOFs and biochar under the condition of 1:1. On the one hand, the presence of SiO_2_ plays a role in forming a bridge between MOFs and biomass, and on the other hand, it can evenly distribute the composite materials, mitigating the volume shrinkage and metal agglomeration caused by high-temperature pyrolysis and enhancing the material’s mechanical properties. This contributes to the formation of a three-dimensional interconnected network structure and an orderly distributed pore structure, which explains the good pore uniformity exhibited by PDC-MIL/BM (1:1). Tarzanagh et al. [19] found that MOFs would react with ethyl orthosilicate, the raw materials for sol-gel preparation. And the addition of MOFs can act as a catalyst to accelerate the formation of gels. This is because the catalytic properties derived from their acidic secondary building units (SBUs), where the metal in the MOFs skeleton acts as the unsaturated coordination center, are functioning as Lewis acid sites [20]. The C-O bond of epoxides (propylene oxide) is activated by coordination with the acidic site on the SBU. Nucleophiles in the reaction system (Cl^−^, H_2_O, NO^3−^, etc.) attack the less hindered carbon atoms on the epoxides, facilitating ring opening and speeding up the consumption of H^+^ in the solution. On the one hand, it can quickly adjust pH. On the other hand, it also plays a role in promoting hydrolysis. The acceleration of hydrolysis increases the rate of cross-linking of the hydrolyzed mixed sol, thereby improving the quality of the gel, as the skeleton remains relatively intact. The gel time is too long, which will lead to undesirable agglomeration, while very short gel time may hinder the growth of the gel’s skeleton. This also explains the reasons for the low bond energy of Si-O bonds at different ratios: with lower MOF proportions, the gel formation rate is slow, and the hydrolysis and condensation reactions are not sufficiently comprehensive, resulting in the incomplete Si-O bond structure. However, with higher MOF proportions, the gel formation rate is too fast, which may lead to local accelerated hydrolysis, followed by polymerization to precipitate, which prevents the non-hydrolyzed sol from forming a uniform gel, thus affecting the growth of the gel’s skeleton. At the same time, the hydrolyzed ethyl orthosilicate will react with the carboxyl groups in the organic ligand skeleton of MOFs, forming an ester functional group. Cross-linking polymerization occurs between MOFs and SiO_2_, thus better integrating the biomass with the MOFs to form interconnected pore channels. Therefore, the Si-O bond absorption peak intensity of PDC-MIL/BM (1:1) material is high, and the BET-specific surface area increases significantly with the increase in MOFs, while in other ratios, the increase in BET surface area is gradual, only increasing by 22.06 m^2^/g from a 3:1 to a 5:1 ratio.

### 3.4. Hg^0^ Removal Performance

#### 3.4.1. Influence of Adsorption Temperature on Mercury Removal Performance of Materials

**Figure 6:**
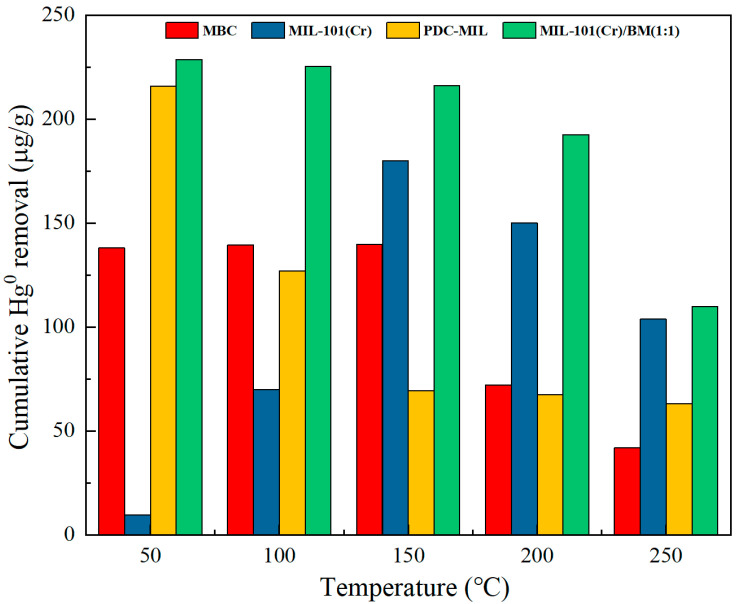
Effect of adsorption temperature on Hg^0^ removal performance.

The effect of increasing the adsorption temperature on the mercury-removal performance of MIL-101(Cr) and its derived composite carbon materials is shown in Figure 6. At 50 °C, 100 °C, and 150 °C, the mercury-removal performance of modified biochar MBC has little difference, reaching 139 μg/g. However, its performance decreases significantly at 200 °C and 250 °C. Some studies have shown that a low temperature is conducive to physical adsorption, and a high temperature is conducive to chemical adsorption. At low temperatures, MBC mainly exhibits a high adsorption capacity for Hg^0^ due to its high mesoporous ratio pore structure. As the temperature increases, chemical adsorption begins to dominate, and the desorption phenomenon accompanied by high temperatures intensifies. MBC maintains a strong adsorption performance for Hg^0^ even when desorption is severe at 150 °C due to its efficient coordination of multiple metal oxides. However, as the temperature increases, desorption continues to intensify, and the removal capacity of Hg^0^ by MBC begins to decrease at 200 °C and 250 °C. When the adsorption temperature increased from 150 °C to 200 °C, the adsorption capacity decreased by 48.44%. As a microporous material, the adsorption capacity of MIL-101(Cr) is low at low temperatures, only 9.72 μg/g. With the increase in temperature, the chemisorption performance of MIL-101(Cr) begins to appear, and the Hg^0^ removal performance of MIL-101(Cr) is higher than that of MBC at 150 °C, 200 °C, and 250 °C. Due to its higher pore richness, the desorption phenomenon at high temperatures was alleviated, and the adsorption capacity only decreased by 16.67% when the temperature increased from 150 °C to 200 °C. The pyrolyzed MOFs material PDC-MIL exhibits similar removal characteristics as MBC, with higher Hg^0^ removal at 50 °C. However, compared with MBC, PDC-MIL has a higher specific surface area and higher mesopore ratio. The Hg^0^ removal performance of PDC-MIL at 50 °C is higher than that of MBC, reaching 215.82 μg/g, which is higher than other MIL-101(Cr)-derived carbon materials [9]. However, the shortcomings of a single metal make the adsorption performance of PDC-MIL poor. Compared with MBC, PDC-MIL has obvious performance decline at 100 °C, with a decrease of 41.14%. However, as the temperature continues to rise, the decrease in PDC-MIL at 200 °C and 250 °C is very small, 2.93% and 6.05%, respectively. This may be because compared with MBC, PDC-MIL has a more abundant pore structure and smaller average pore size, resulting in a lower escape rate for Hg^0^ desorption. Moreover, with the increase in temperature, the thermal diffusion effect of Hg^0^ in the material is intensified, and the abundant pore structure and small average pore size increase the contact probability of Hg^0^ with the active site in the pore, thus accelerating the reaction rate. In summary, in order to obtain high-performance Hg^0^ adsorbents in a wide temperature range, it is necessary to have excellent pore structure, and at the same time, it is necessary to cooperate with multiple metals to improve the adsorption performance of the material at high temperatures. PDC-MIL/BM (1:1) has both a large specific surface area and an excellent pore structure, as well as the synergistic effect of multiple metals. Therefore, it maintains a high Hg^0^ adsorption capacity in a wide temperature range. It not only has a 192.56 μg/g adsorption capacity at a high temperature of 200 °C but also decreases by only 10.91% when the temperature increases from 150 °C to 200 °C. The composite materials prepared by the sol-gel method not only maintain the excellent pore structure of PDC-MIL but also introduce multiple metals to improve the adsorption performance. At the same time, the participation of biomass also reduces the direct cost of mercury pollution treatment, making mercury pollution control technology more economical and practical.

#### 3.4.2. Effect of Composite Ratio on Mercury Removal Performance of Composite Materials

**Figure 7:**
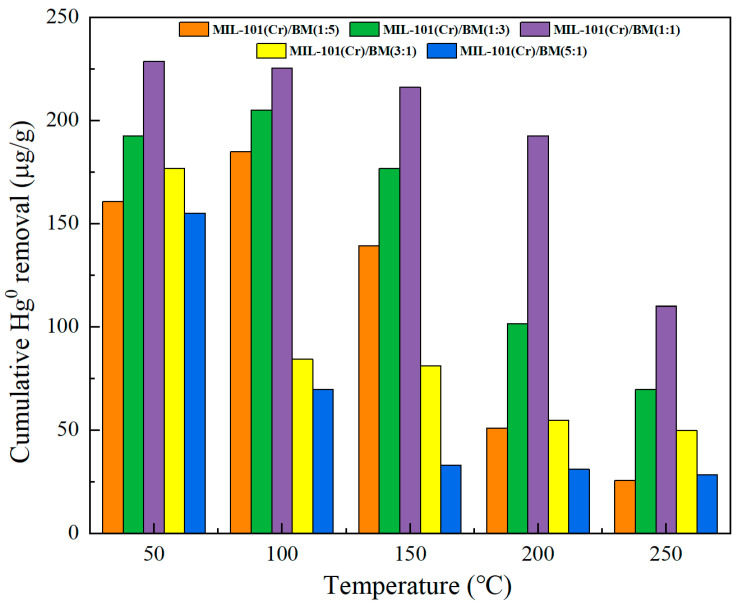
Effect of composite ratio on Hg^0^ removal performance.

Figure 7 shows the effect of pyrolysis of MIL-101 (Cr) and biomass at different ratios on the Hg^0^ removal performance. As the ratio of MIL-101 (Cr) to biomass increases from 1:5 to 1:1, the mercury removal performance of the composite materials improves over the entire temperature range. This is because as the proportion of MOFs increases, the pore structure of the composite materials is optimized. Materials with excellent pore structures can meet the physical adsorption of Hg^0^ at low temperatures, while at high temperatures, the desorption of Hg^0^ can be weakened (escape rate reduced), meeting the collision probability required for Hg^0^ to contact active sites. The addition of multiple metals also enhances the material’s oxidation ability at high temperatures. However, as the proportion of MOFs continues to increase, the material’s removal performance of Hg^0^ in the entire temperature range decreases, mainly because MOFs act as a catalyst in the process of forming sol-gel. An excessive number of MOFs accelerate the formation of gel, and the growth of the skeleton structure of gel is affected. Although the specific surface area of the composite improves due to the increased proportion of MOFs, the pore structure becomes poor, and because the gel time is too fast, metal oxides and MOFs appear as an agglomeration or even precipitation. With the increase in the ratio of MOFs, the specific surface area of the composites increases slowly, remaining lower than 16.25%, which is the value when changing from a ratio of 1:3 to 1:1. In conclusion, although the composites prepared by the sol-gel method can achieve uniform mixing of multi-component systems, thus improving the homogeneity and performance of the materials, the catalytic effect of MOFs will affect the process of gel formation, thus affecting the cross-linking density, porosity structure, and functionalization degree of the composites. Therefore, an appropriate proportion of MOFs is crucial for improving the Hg^0^ removal performance of composite materials. In order to enhance the performance of MOF composite materials, precise synthesis control and comprehensive performance evaluation must be carried out to ensure that the material’s characteristics reach their optimal conditions.

## 4. Conclusions

1. By using the sol-gel method, MIL-101(Cr) and biomass were combined in different proportions and doped with multiple metal ions, and high performance Hg^0^ adsorbent was prepared in a wide temperature range. Among them, PDC-MIL/BM (1:1) has excellent pore structure and good pore homogeneity in the material, which makes the physical adsorption performance of the material good at a low temperature and also reduces the desorption of Hg^0^ caused by a high temperature, thus enhancing the adsorption performance of the material at high temperatures. The cumulative adsorption capacity of Hg^0^ reached 192.56 μg/g at 200 °C for 3 h;
2. MIL-101(Cr) will act as a catalyst that influences the gel formation process. When the proportion of MOFs is small, the gel formation proceeds slowly, leading to the particles’ aggregation in the sol, which compromises the uniformity and dispersion of the material. However, a high proportion of MOFs accelerates the gel excessively, impacting the growth of the gel’s skeletal structure and resulting in the uneven internal structure of the material. When the ratio of the two is 1:1, the gel skeleton develops effectively, and SiO_2_ is well dispersed between the MOFs and biochar. On the one hand, the existence of SiO_2_ plays a role in forming a bridge between the MOFs and biomass; on the other hand, it can facilitate the distribution of the composite material. It mitigates the volume shrinkage and metal agglomeration caused by high temperature pyrolysis, enhances the mechanical properties of the material, and promotes the formation of a three-dimensional interconnected network and a uniformly distributed pore structure;
3. Pore structure and active sites are the key factors affecting the adsorption performance of Hg^0^. Excellent pore structure can reduce the desorption of Hg^0^ caused by high temperatures on the one hand and increase the contact probability between Hg^0^ and active sites on the other hand so that the material can exhibit excellent Hg^0^-removal characteristics at both low and high temperatures. The metal ions introduced in the composite process and the excellent pore structure formed by the coupling of MOFs and biochar together constitute the efficient adsorption performance of the composites in a wide temperature range.

## Figures and Tables

**Table 1:** Pore structure parameters of samples.

Sample	BET Specific Surface Area (m^2^/g)	Pore Volume (cm^3^/g)	Average Pore Size (nm)	Relative Specific Pore Volume (%)		
				Microporous	Mesoporous	Macroporous
MIL-101(Cr)	2438.67	1.236	2.50	60.88	29.68	9.44
MBC	107.76	0.112	11.20	2.87	64.12	33.01
PDC-MIL	333.39	0.348	5.56	4.33	84.89	10.78
PDC-MIL/BM(1:5)	192.19	0.163	8.57	3.91	63.39	32.70
PDC-MIL/BM(1:3)	225.05	0.210	8.18	4.05	66.63	29.32
PDC-MIL/BM(1:1)	268.72	0.231	7.59	5.16	68.42	26.42
PDC-MIL/BM(3:1)	284.11	0.251	6.44	8.61	67.54	27.85
PDC-MIL/BM(5:1)	306.17	0.277	6.06	9.53	64.27	26.20

## Data Availability

The data that support the findings of this study are available from the corresponding author upon reasonable request.
